# Detection of Coronary Artery and Aortic Arch Anomalies in Patients with Tetralogy of Fallot Using CT Angiography

**DOI:** 10.3390/jcm11195500

**Published:** 2022-09-20

**Authors:** Zsófia Kakucs, Erhard Heidenhoffer, Marian Pop

**Affiliations:** 1 Mures County Clinical Emergency Hospital, 540136 Targu Mures, Romania; 2 Clinical County Hospital Mures, 540103 Targu Mures, Romania; 3 ME1 Department, “George Emil Palade” University of Medicine, Pharmacy, Science and Technology of Targu Mures, 540142 Targu Mures, Romania; 4 Emergency Institute for Cardiovascular Disease and Transplant of Targu Mures, 540136 Targu Mures, Romania

**Keywords:** CT angiography, Fallot tetralogy, coronary arteries, aortic arch anomalies

## Abstract

**Background:** Tetralogy of Fallot (TOF) is the most common form of cyanotic congenital heart disease (CHD). Furthermore, the prevalence of anomalous origin of a coronary artery is higher in patients with TOF than in the general population (6% vs. ≤1%). Preoperative assessment of cardiovascular anatomy using computed tomography (CT) angiography enables the adaptation of the surgical approach to avoid potentially overlooked anomalies. Our purpose was to determine the prevalence of coronary artery and aortic arch anomalies in a cohort of TOF patients. **Methods:** In this retrospective analysis, data were collected from CT reports (2015–2021) of 105 TOF patients. All images were acquired using a 64-slice multi-detector CT (MDCT) scanner. **Results:** The median age of the patients was 38.7 months, with a male-to-female ratio of 1.39. The overall prevalence of coronary artery anomalies (CAAs) was 7.61% (8 of 105 cases). The anomalous origin and course of coronary arteries across the right ventricular outflow tract (RVOT; prepulmonic course) were defined in 5.71% of cases (six patients). In four of these, the left anterior descending artery (LAD) originated from the right coronary artery (RCA), while in two cases, the RCA arose from the LAD. In the remaining two patients, the coronary arteries followed an interarterial course. The most frequent anomalous aortic arch pattern in the overall TOF population was the right aortic arch (RAA) with mirror image branching, seen in 20% of patients (21 cases). The most frequent anomaly of the supra-aortic trunks was bovine configuration, found in 17.14% (18 cases). **Conclusions:** The prevalence of CAAs and aortic arch anomalies detected by CT angiography was in line with the data reported in anatomical specimens. Therefore, this technique represents a powerful tool for the evaluation of congenital cardiovascular anomalies.

## 1. Introduction

**Figure 1:**
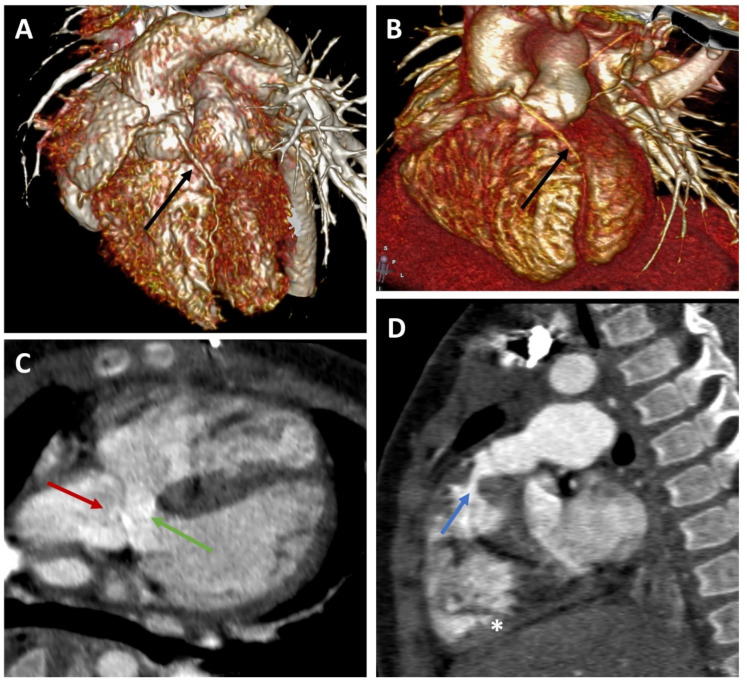
Representative CT angiography images. (**A**,**B**) Volume-rendering technique (VRT) reconstructions for CT images of 2 patients with Fallot tetralogy (ages: 7 and 14 months). Black arrow: anomalous course of the left anterior descending artery over the right ventricle outflow tract (RVOT). (**C**,**D**) Double oblique reconstruction of CT images in an infant with Fallot tetralogy. Red arrow: Mildly dilated aortic root overlapping the interventricular septum. Green arrow: Ventricular septal defect. Blue arrow: Narrowing of the right ventricular outflow tract (subpulmonary stenosis). The right ventricle (star) is mildly hypertrophied.

Tetralogy of Fallot (TOF) is the most common form of cyanotic congenital heart disease (CHD), consisting of a ventricular septal defect, stenosis, or atresia of the pulmonary outflow; biventricular origin of the aorta; and right ventricular hypertrophy as a secondary feature (Figure 1C,D). This combination of defects occurs in 421 cases per million live births, constituting around 7–10% of all congenital cardiac malformations [1,2]. Echocardiography is the initial modality of choice for making the diagnosis and follow-up. Useful secondary diagnostic tools are electrocardiogram (ECG) and chest radiography. Findings from these tests are often suggestive but not definitive for the diagnosis of TOF. Invasive angiography is sometimes needed to establish the diagnosis and to provide detailed anatomy and hemodynamic characterization. As a good alternative to invasive cardiac catheterization, multi-detector computed tomography (MDCT) with high spatial and temporal resolution plays an important role in the evaluation of complex anatomical findings [3].

Coronary artery anomalies (CAAs) are a diverse group of congenital conditions with highly variable clinical presentation and pathophysiological mechanisms usually observed in the context of complex CHDs [4]. The prevalence of the anomalous origin of a coronary artery is higher in patients with TOF than in the general population (6% vs. ≤1%), as described by Koppel et al. [5].

Right aortic arch (RAA) anomalies are known to occur in association with cardiac outflow malformations, of which the most common type is TOF [6]. The prevalence of RAA varies between 13% and 34% in TOF patients, as opposed to 0.1% in the general population [7,8]. While the appearance of CAAs and RAA in TOF has been intensively analyzed, the number of studies describing the variety of branching patterns in patients with the left aortic arch (LAA) configuration is limited.

Preoperative identification of the coronary tree and aortic arch anatomy in patients with TOF is relevant to enable the adaptation of the surgical approach and avoid potentially overlooked anomalies. Some cases of CAAs crossing the right ventricular outflow tract (RVOT) are not detectable intraoperatively due to their intramyocardial course, overlying epicardial fat, or pericardial–epicardial adhesions from previous palliative surgery [9].

Consequently, preoperative computed tomography (CT) angiography may be essential in coronary assessment to minimize the risk of negative postoperative outcomes such as myocardial infarction and patient death. Furthermore, knowledge of aortic arch morphology is of crucial importance in deciding the appropriate access to palliative interventional procedures (e.g., constructing a systemic to pulmonary artery shunt), placement of monitoring lines, and cannulation for extracorporeal life support [7].

In the present study, our goal was to determine the prevalence of coronary artery and aortic arch anomalies in a cohort of TOF patients and to compare our results with findings from previous studies. The majority of studies were based on invasive coronary angiography (ICA) for assessing coronary anatomy; therefore, we also aimed to highlight the role of CT angiography in preoperative evaluation.

## 2. Materials and Methods

This retrospective analysis included 105 TOF patients who underwent CT angiography examinations between 2015 and 2021 for the evaluation of cardiovascular anatomy at a single tertiary care hospital. All examinations were performed using a 64-MDCT scanner (Definition AS, Siemens, Erlangen, Germany or Revolution HD, GE Healthcare, Milwaukee, WI, USA) using ECG gating and power injectors, with contrast volumes and flow rate according to local protocols.

**Figure 2:**
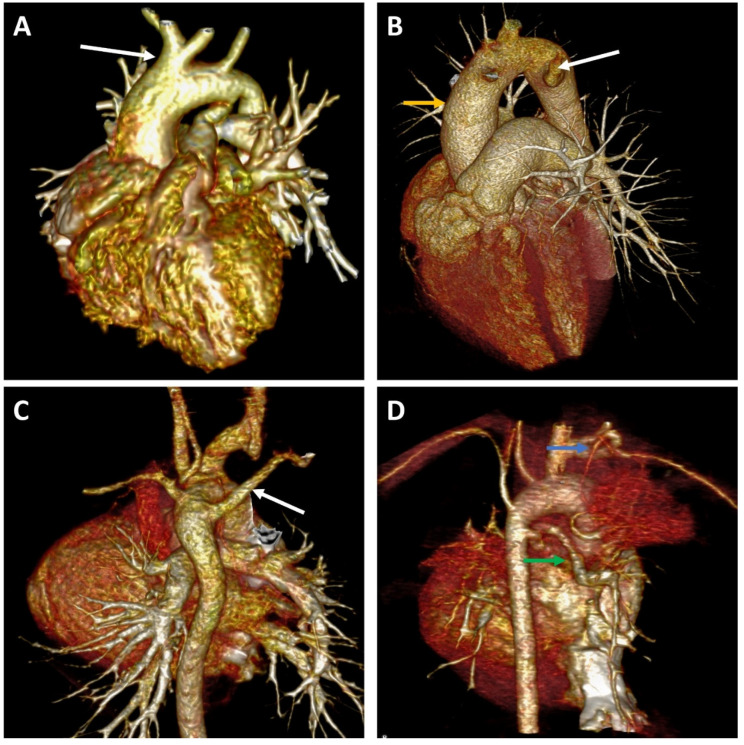
Representative image for aortic arc anomalies. (**A**) Left-sided aortic arch with left carotid artery (white arrow) from brachiocephalic trunk (“bovine arch”). (**B**) Right-sided aortic arch (yellow arrow) with anomalous left subclavian artery (white arrow). (**C**) Left-sided aortic arch with aberrant right subclavian artery (white arrow). (**D**) Major aortopulmonary collateral arteries (MAPCAs) feeding the left pulmonary artery (green arrow) and the apical portion of the left upper lobe (blue arrow).

The institutional reports database was queried to identify Fallot tetralogy patients who underwent CT examinations, and the reports were further analyzed. All reports were provided by a radiologist with 5+ years of experience in cardiovascular imaging (EACVI Level 3). The data were collected retrospectively from the CT report protocols, with variables being recorded in an MS Excel database (see Figure 1 and Figure 2 for representative images). We included all patients with a diagnosis of TOF, including those with extreme variants (pulmonary atresia with major aortopulmonary collateral arteries (MAPCAs) or Fallot type double outlet right ventricle (DORV)). For patients with multiple examinations, the data were recorded only once. We recorded the following variables: gender, age, thoracic vessels anatomical course, and variants, as well as coronary arteries’ course. CAAs and aortic arch anomalies were assessed in both unrepaired and repaired TOF patients. This is a small sample size and a single-center study; therefore, neonates, children, and adults were also included. Patients who underwent surgical repair in our center before 2015 were evaluated preoperatively through ICA as the golden standard. In this subgroup, CT angiography was executed as part of a preoperative screening protocol for later interventions if any acute cardiovascular event or late postoperative TOF complication arose. With the emergence of newer-generation CT-scanners and software, CT angiography was preferred to ICA due to its non-invasive approach, and, as such, it was the predominantly used evaluation method, preceding primary TOF repair after 2015, in our center.

From these data, the incidences of both surgically critical (prepulmonic course) and non-critical CAAs and aortic arch anomalies were calculated.

All statistics were performed using GraphPad InStat. Associations were tested using Chi-test with a significance level *p* < 0.05. The Emergency Institute for Cardiovascular Diseases and Heart Transplant ethics committee approved the tertiary analysis of data through address 8984/2020.

## 3. Results

In this study, a comprehensive CT angiography evaluation was performed on 105 TOF patients, of whom 61 (58.1%) were male (male/female ratio of 1.39). The median age of this population was 38.7 months (interquartile range 6.9–179.4), with a broad age distribution ranging from 0 months to 47 years. The majority of participants were under 18 years of age, and more than one-third were neonates and infants (35.2%). Table 1 summarizes the demographic characteristics of these patients.

The overall prevalence of CAAs was 7.61% (8 of 105 cases). Anomalous origin and course of the coronary arteries across the RVOT (prepulmonic course) were defined in 5.71% of cases (six patients), representing 75% of CAAs. Within this group, the left anterior descending artery (LAD) emerging from the right coronary artery (RCA) was found in four patients (Figure 1A,B), while RCA originating from the LAD was seen in two cases. In the remaining two patients, the CAAs followed an interarterial course, with one case of LAD arising from the RCA and one case of RCA originating from the left main coronary artery (LM). Additional anatomical findings were noted, namely four other cases with a prominent conus artery similar in caliber to the RCA (Table 2).

Of 105 patients with TOF, CAAs following a prepulmonic course were found in five patients in addition to aortic arch anomalies. Of these, LAD emerging from the RCA and bovine arch was observed in three patients, while LAD originating from the RCA and RAA with mirror image branching was seen in one. Another patient had RCA arising from the LAD in addition to RAA with mirror-image supra-aortic trunks. No statistically significant association was found between the prevalence of CAAs and anomalous aortic arch patterns (*p* > 0.9999).

The variation in aortic arch position and branching pattern is summarized in Table 3 and Table 4. In total, 76.2% of patients (80) had LAA, while RAA was seen in 23.8% of cases (25 patients). As determined using the classification of Popieluszko et al. [10]—see Table 3—the most common aortic arch pattern among these patients was type 1 (normal configuration), which occurred in 40.95% of all cases. The second most frequently observed configuration was RAA (23.8%), followed by the bovine arch variant (16.19%; Figure 2A). Equal proportions of aberrant branching of the left vertebral artery (LV) and right subclavian artery were observed (2.85%; Figure 2C). Only one patient had both a bovine arch and an aberrant LV branching pattern (0.95%).

During the evaluation of LAA patterns, we found three unclassified arch anatomy features. In one patient, both the left common carotid artery (LCC) and left subclavian artery (LSA) arose from the anterior aspect of the aortic arch, while in another patient, only the LCC had an anterior origin. A separate origin of each supra-aortic trunk was also noted in one case. None of the patients in this series had a type 5 (common carotid trunk) branching configuration.

In Table 4, we present the various forms of RAA. Within this group, 84% (21 of 25 patients) had RAA with mirror image branching of the main vessels, 12% (three patients) had RAA with an aberrant left subclavian artery (ALSA; Figure 2B), and 4% (one patient) had RAA with a bovine arch. Three types of RAA exist in the literature, as defined by the Edwards classification scheme [11,12]. However, none of the CT scans showed obliteration or isolation of the LSA with collateralization (type III).

The most frequent anomalous aortic arch pattern found in the overall TOF population was RAA with mirror image branching, identified in 20% of cases (21 patients). The most frequent anomaly of the supra-aortic trunks was bovine configuration, found in 17.14% of cases (eighteen patients, one of them in association with RAA), followed by an aberrant course of the subclavian artery in 5.71% of cases (six patients, equal numbers of left and right subclavians).

Other vascular anomalies were detected using CT angiography: patent ductus arteriosus (PDA; eight patients, 7.61% of all cases), major aortopulmonary collateral arteries (MAPCAs; four patients, 3.8%; Figure 2D), prominent sinoatrial nodal artery (one patient, 0.95%), and ductal diverticulum (one patient, 0.95%). PDA was associated with RAA in two cases, while the remaining six patients had a left-sided aortic arch (Table 5).

## 4. Discussion

The survival rate of neonates with TOF has increased over the years due to highly sophisticated imaging techniques and successful management of patients with CHDs. The treatment strategies currently used in TOF have improved, offering excellent long-term survival (30-year survival rate of 68.5% to 90.5%). However, reintervention procedures are still often required [13]. Diagnostic imaging findings provide key information about the anatomical relationships of cardiac and extracardiac structures and hemodynamic features prior to surgical repair. The goals of preoperative imaging in TOF are to establish the severity of the primary anatomical lesions and the degree of functional alterations, as well as identify associated anomalies (such as variants of aortic arch patterning, CAAs, PDA, and MAPCAs). Moreover, the assessment of the presence and extent of lesions is required to determine the optimal timing and approach of surgical interventions—either definitive early repair or a palliative interventional procedure followed by surgery at a later stage [14].

Inadvertent division of coronary vessels crossing the RVOT during right ventriculotomy or transannular repair can lead to serious complications. Thus, clear delineation of the origin and course of these arteries is vital for the selection of a suitable surgical approach. Alternative surgical techniques must be chosen individually for this subset of anomalies (e.g., transatrial-transpulmonary repair, RVOT stenting) [15,16]. Cases have been described where a CAA crossing the RVOT was not identified during the preoperative assessment and did not become clear during surgical repair, causing it to be damaged, followed by myocardial infarction [5].

Different methods of visualization of the coronary tree anatomy can be used during the surveillance of patients with TOF. ICA was considered the gold standard imaging modality to identify and classify CAAs. However, a potential complicating factor during ICA examination is the counterclockwise rotation of the aortic root, which causes overlapping of the right and left coronary arteries in anteroposterior projection [17]. The possible utility of ICA has been reappraised, and this technique is being progressively replaced by coronary computed tomography angiography (CCTA). As CCTA is non-invasive, it is more widely applicable for diagnosis [4].

Multi-detector CCTA offers a detailed characterization of cardiac structures and small vessels using static images as well as 3D reconstructions. Although no studies have compared coronary angiography and CT scanning directly, CCTA has been shown to be a promising substitute for ICA as a method of identifying aberrant coronary patterns [5]. The main limitations of CT angiography are the dose of ionizing radiation given to the patients and the potential induction of contrast-induced nephrotoxicity. However, the patient’s radiation dose is higher during ICA when an appropriate pediatric protocol is used [18]. Moreover, invasive angiography also involves the administration of an intravascular contrast agent, leading to iatrogenic renal injury in susceptible individuals. It is important to note that the introduction of ECG gating and postprocessing techniques has resulted in significant improvements in image resolution, as well as reductions in acquisition time and radiation dose [4].

In adults, cardiac magnetic resonance (CMR) imaging has an established role in the diagnosis of CAAs. However, this method has had limited success in the evaluation of children as the smaller heart size and faster heart rate in pediatric patients lead to poor spatial and temporal resolution. Moreover, additional sedation may be needed for adequate image quality. In a recent study, CMR angiography did not perform well in patients younger than 4 months; diagnostic image quality was obtained in only 17% of cases, even though all examinations were performed under general anesthesia [19].

There is also increasing evidence for the successful identification of anatomical evidence by transthoracic echocardiography, which can be a suitable method of primary investigation in children that avoids radiation exposure. However, echocardiography is not routinely used for the visualization of coronary vessels in the adult population because of its lower spatial resolution and suboptimal acoustic windows [4].

Due to many possible anatomic variants, which are not considered anomalies, the term CAA, as a definition, has historically been restricted to those occurring in less than 1% of the general population [4]. The reported prevalence of CAAs in patients with TOF is between 2% and 23%. The overall prevalence of these congenital malformations was 6% in a meta-analysis including 28 studies, which was similar to our results (7.61%). In our TOF patients, the proportion of surgically significant anomalies with coronary arteries passing across the RVOT was 75% of all CAAs; again, this was similar to the percentage (72%) calculated in the previously mentioned large-scale meta-analysis [5]. Anomalous origin of the LAD from the RCA or the right sinus of Valsalva was the most common CAA in the majority of studies; this was also the vascular anomaly most frequently encountered in conjunction with prepulmonic course in TOF patients [5,14]. Our results correlated with these findings.

Solitary coronary arteries arising from one of the sinuses of Valsalva are also frequently reported CAAs in patients with TOF [9,17,20]. The prevalence varies between 0.0240% and 0.066% in the general population, whereas these anomalies are reported between 1.5% and 3.7% in TOF patients [21,22]. In the present study, a single coronary artery was found in three cases (2.85%), which is in line with the reported frequency in TOF patients. This type of CAA may cause myocardial ischemia by different mechanisms, even in the absence of atherosclerotic coronary lesions. The abnormal vessel angulations or courses may affect the distribution of blood flow, leading to serious complications. Coronary classification systems are useful tools for providing detailed information about these anomalies; however, neither of them can be applied in all TOF cases. The Leiden Convention coronary coding system has been shown to be a feasible method for the characterization of single coronary arteries in the setting of complex CHD; however, this classification is not routinely used in patients with TOF. In this classification system, the aortic sinuses are described as left- or right-facing or non-facing relative to the pulmonary valve sinuses; therefore, the applicability is limited in cases of pulmonary atresia. In such cases, a detailed description of the coronary anatomy, as well as associated characteristics, should be provided for treatment planning [23].

By the classic definition, five CAA course subtypes exist: prepulmonic, interarterial, subpulmonic (intraconal or intraseptal), retroaortic, and retrocardiac [24]. As in the general population, not all variations in CAAs are of equal clinical importance. Coronary arteries following an interarterial course (between the ascending aorta and the pulmonary trunk) with high-risk anatomic features (e.g., intramural tract, slit-like ostium) are considered malignant and require additional corrective surgery. In this study, the morphological aspects of these arteries were deemed benign.

It should also be noted that a single coronary artery with an interarterial course may increase the risk of major adverse cardiac events [4]. In the case of subpulmonic course, the coronary vessel exits the aorta below the pulmonic valve and traverses the RVOT, pulmonary infundibulum, and interventricular septum. The differential diagnosis between the intraseptal and the intramural interarterial course of a coronary artery is especially relevant and can be difficult; however, an intraseptal CAA has a more inferior position [25]. In this study, none of the CT scans showed CAAs with subpulmonic, retroaortic, or retrocardiac courses in patients with TOF. In our sample population, represented mainly by preoperative TOF patients with a median age of 3 years, the clinical impact of the “malignant” variant remains to be investigated in follow-up examinations.

The conus artery is a small branch that usually originates from the proximal portion of the RCA. A large conus artery (as identified in four cases in this study) may supply a larger area of myocardium and therefore should undoubtedly be preserved during corrective surgery [26]. Variants larger than the RCA have been described, crossing over the outflow tract of the right ventricle and reaching the heart apex. These variants could be easily mistaken for an accessory LAD on ICA examination in the anteroposterior projection [27]. Hence, the true prevalence remains uncertain [5].

Congenital aortic arch malformations embody a large group of anomalies that result from the disordered embryogenesis of the pharyngeal arches, including abnormal or incomplete regression of one or more embryogenic vascular segments [28]. The recognition of these aberrations is of paramount importance in TOF patients to ensure accurate preoperative surgical decisions. In this study, the classic branching pattern of the aortic arch (left-sided aorta giving rise to the brachiocephalic trunk, LCC, and LSA) was found in 40.95% of patients, whereas the prevalence of this pattern is significantly higher in the general population (80.9%) [10]. This finding suggests that the occurrence of atypical aortic arch patterns is higher in TOF patients. According to the Popieluszko classification, the most frequently seen anomalous arch pattern in our patients was the RAA variant (23.8%). The incidence of RAA has been reported as around 25% in previous studies [7,18,29]. Apart from the normal branching pattern, the most common left-sided aortic arch pattern was the bovine arch, including the common origin of the innominate artery and the LCC, which were seen in 40.95% and 16.19% of patients, respectively. These findings are in accordance with those of Moustafa et al. (40.4% and 22.8%) [29] and Tawfik et al. (36% and 16%) [30].

The aberrant origin of one of the subclavian arteries occurs with a higher incidence in patients with TOF than in the general population, where it is less than 2% [31,32]. Aberrancy of the subclavian artery can influence monitoring line placement—if this anomaly is recognized preoperatively, the arterial line should be placed in the contralateral radial or the femoral artery [33,34]. Different palliative procedures are offered depending on clinical presentation, although the procedure most often carried out remains the modified Blalock–Taussig shunt, which involves inserting an interposed graft between the subclavian artery and the ipsilateral pulmonary artery [35]. Failure to identify an aberrant subclavian artery prior to surgery might lead to incorrect insertion of the shunt between the carotid artery and the pulmonary circulation [7].

Within the RAA branching configuration, a mirror image of supra-aortic trunks was observed in 84% of patients, whereas RAA with ALSA was found in 12% of cases. These findings are in accordance with the results of Prabhu et al., who identified RAA with mirror image branching in 86.6% of 688 patients and RAA with ALSA in 10.6% [7].

Fallot-type DORV (where DORV means abnormal ventriculoarterial connection with both great vessels arising, entirely or predominantly, from the right ventricle, and Fallot-type means that DORV mimics elements of TOF) is an uncommon complex congenital heart anomaly in which early complete repair should be considered to avoid ventricular volume overload and progression of tricuspid valve regurgitation. The complex anatomy of Fallot-type DORV can make surgical correction a challenge; therefore, comprehensive preoperative assessment and planning are needed [36].

Tetralogy of Fallot with pulmonary atresia (TOF-PA) is a severe variant of TOF, characterized by a lack of antegrade flow into the pulmonary arteries. To provide additional blood flow to the pulmonary circulation, PDA is usually seen in association with TOF-PA. Stenting of PDA has gained acceptance for palliation in TOF-PA, although the PDA is usually elongated and tortuous, making the implantation of a rigid, straight stent challenging. Hence, advanced imaging with MDCT angiography is necessary for case selection and preprocedural planning [37]. The common carotid and axillary artery approaches are the most feasible routes for stent implantation. However, ductal stenting is contraindicated in the case of the common carotid trunk, as damage to the vessels during direct surgical or percutaneous approach could lead to neurological deficits [7].

Finally, MAPCAs are persistent tortuous fetal arteries branching from the aorta or systemic arteries that form in an attempt to compensate for the underdeveloped pulmonary circulation via multifocal supply. TOF-PA with MAPCAs is a severe and rare type of congenital heart defect [38]. We found this malformation in just 5% of cases. Surgical management to restore normal pulmonary blood flow is challenging due to the heterogeneity of arborization in these patients [35]. Therefore, preoperative imaging is necessary to achieve a complete understanding of cardiovascular anatomy.

The systematic use of CT angiography for preoperative identification of CAAs and aortic arch patterns in TOF was scarcely reported. Therefore, one of the main objectives of our study was to highlight the role of CT angiography in the evaluation of complex anatomical findings, as a good alternative to invasive cardiac catheterization. Moreover, the number of studies describing the variety of aortic arch and branching patterns in TOF patients is limited, and most provide incomplete information regarding the entire spectrum of aortic anomalies. This led to our aspiration to encompass all classifiable and non-classifiable aortic malformations in this specific population.

## 5. Study Limitations

There are several limitations to the design of this study. First, this is a single-center, small-sample observational study based on retrospective data analysis. Therefore, no comparison of diagnostic accuracy was performed for CT angiography and ICA in TOF patients. As mentioned previously, no prospective studies comparing these diagnostic imaging methods have been found in the literature [5]. However, Gorenoi et al. declared that CCTA with at least 64 slices should be used to identify coronary alterations in order to avoid invasive investigation in patients with CHD [39].

Second, the concordance of coronary artery patterns between the preoperative CCTA and corrective surgical findings was not evaluated. However, Goo et al. demonstrated that dual-source CCTA with a high temporal resolution was useful for the assessment of coronary artery anatomy before corrective surgery in TOF, exhibiting not only a high concordance rate (95.0%) with surgical findings but also a high level of diagnostic accuracy (96.9%) [9].

Technical limitations were also present. Not only was it possible that different contrast agents might have impacted the quality of the CT examination [40], but the results of this study were obtained using a 64-slice last-generation CT scanner. In order to reduce the number of misinterpreted cases, higher temporal resolution CCTA is needed. The second-generation 128-slice dual-source CT is one of the most frequently used imaging modalities for evaluating TOF patients, enabling high temporal resolution and scanning speed, as well as a low radiation dose. Moreover, diagnostic accuracy is expected to be further improved with the recently introduced third-generation 192-slice dual-source CT system, which provides even better image quality at lower radiation doses [41].

## 6. Conclusions

The prevalence of CAAs and aortic arch anomalies detected by CT angiography in this study is in line with the data reported in anatomical specimens. Therefore, CT angiography represents a powerful tool for the evaluation of congenital cardiovascular anomalies.

## Figures and Tables

**Table 1:** Demographic characteristics.

Gender	Male	58.1% (61/105)
	Female	41.9% (44/105)
	M:F ratio	1.39
Age distribution	Range	0 month to 47 years
	Median	38.7 months (IQR 6.9–179.4)
	<1 year	35.2% (37/105)
	1–18 years	47.6% (50/105)
	≥18 years	17.1% (18/105)

IQR = interquartile range (IQR); M:F ratio = male to female ratio.

**Table 2:** Coronary artery anomalies and prominent conus artery.

Course	Origin	Overall Prevalence
Prepulmonic	LAD from RCA	3.8% (4/105)
	RCA from LAD	1.9% (2/105)
Interarterial	LAD from RCA	0.95% (1/105)
	RCA from LM	0.95% (1/105)
**Other coronary findings**	Coronary pattern	Overall prevalence
	Prominent conus artery	3.8% (4/105)

LAD = left anterior descending artery; LM = left main coronary artery; RCA = right coronary artery.

**Table 3:** Different types of aortic arch variations.

	Prevalence in LAA	Overall Prevalence	Characteristics
**Popieluszko classification**			
Type 1—normal	53.75% (43/80)	40.95% (43/105)	-
Type 2—bovine arch	21.25% (17/80)	16.19% (17/105)	One patient had PDA
Type 3—LV from aortic arch	3.75% (3/80)	2.85% (3/105)	-
Type 4—bovine arch and LV	1.25% (1/80)	0.95% (1/105)	-
Type 5—common carotid trunk	-	-	-
Type 6—ARSA	3.75% (3/80)	2.85% (3/105)	-
Type 7—RAA	-	23.8% (25/105)	-
**Unclassified branching pattern**			
LCC from anterior aspect of aortic arch	1.25% (1/80)	0.95% (1/105)	-
LCC and LSA from anterior aspect of aortic arch	1.25% (1/80)	0.95% (1/105)	The patient had MAPCAs
RSA from aortic arch	1.25% (1/80)	0.95% (1/105)	-

ARSA = aberrant right subclavian artery; LAA = left aortic arch; LCC = left common carotid artery; LSA = left subclavian artery; LV = left vertebral artery; MAPCAs = major aortopulmonary collateral arteries; PDA = patent ductus arteriosus; RAA = right aortic arch; RSA = right subclavian artery.

**Table 4:** Types of RAA according to the Edwards classification.

	Prevalence in RAA	Overall Prevalence	Characteristics
Type I—RAA with mirror image	84% (21/25)	20% (21/105)	Two patients had PDA
Type II—RAA with ALSA	12% (3/25)	2.85% (3/105)	-
Type III—Isolated LSA	-	-	-
Unclassified RAA with bovine arch	4% (1/25)	0.95% (1/105)	-

ALSA = aberrant left subclavian artery; LSA = left subclavian artery; PDA = patient ductus arteriosus; RAA = right aortic arch.

**Table 5:** Other vascular findings.

	Prevalence in LAA	Overall Prevalence	Characteristics
PDA	7.5% (6/80)	7.61% (8/105)	One patient had bovine arch
MAPCAs	5% (4/80)	3.8% (4/105)	One patient had LCC and LSA arising from the anterior aspect of the arch
Prominent sinoatrial nodal artery	1.25% (1/80)	0.95% (1/105)	-
Ductal diverticulum	1.25 (1/80)	0.95% (1/105)	-

LAA = left aortic arch; LCC = left common carotid artery; LSA = left subclavian artery; MAPCAs = major aortopulmonary collateral arteries; PDA = patent ductus arteriosus.

## Data Availability

Data are available upon reasonable request.
